# Preliminarily Study on Hydroxyproline Content of Purple-spotted Bigeye (*Priacanthus tayenus*) Scaly Skin and Its Gelatine Quality

**DOI:** 10.21315/tlsr2025.36.1.6

**Published:** 2025-03-30

**Authors:** Widiyanto, Uju, Sitti Hardiyanti Rachman, Mala Nurilmala

**Affiliations:** 1 Department of Aquatic Product Technology, Faculty of Fisheries and Marine Science, Bogor Agricultural University. Dramaga, Bogor, West Java 16680, Indonesia; 2 Ministry of Marine Affairs and Fisheries of the Republic of Indonesia, Medan Merdeka Timur Gambir Central Jakarta 10110, Indonesia; 3 Study Program of Fisheries Product Technology, Faculty of Fisheries and Marine Science, Lambung Mangkurat University, A. Yani, Banjarbaru, South Kalimantan 70714, Indonesia

**Keywords:** Fish Scaly Skin, Gelatine, Halal, Hydroxyproline, Purple-spotted

## Abstract

The investigation of alternative raw materials for gelatine production from fishery industry by-products has gained attention due to the increasing demand for gelatine and the importance for sustainable practices. This study aims to determine the optimal hydrochloric acid (HCl) concentration for mineral removal during pre-treatment, assess hydroxyproline content at various processing stages and characterise the resultant gelatine. The methodology involved pre-treatment of the materials with 0.1 M sodium hydroxide (NaOH) to remove non-collagen proteins, followed by mineral extraction using varying HCl concentrations (0.25, 0.5, 0.75 and 1 M). The process included swelling in 0.2% citric acid for 12 h and gelatine extraction at 65°C for 7 h. The results indicated that 0.25 M HCl was most effective for mineral removal. The hydroxyproline analysis showed an insignificant increase (0.088 mg/mL–0.103 mg/mL) from the pre-treatment stage to the final gelatine product. The physicochemical properties of the liquid gelatine, including yield (6.5 ± 0.39%), pH (6.55 ± 0.11), and gel bloom strength (174 ± 8.54 blooms) conformed to Gelatin Manufacturers Institute of America (GMIA). Functional groups confirmed the presence of gelatine-specific, such as amides A, B, I, II and III. The molecular profile is comparable to commercial gelatine, with α1 chains at 130 kDa, α2 chains at 115 kDa, and β chains at 235 kDa. The gelatine derived from the scaly skin of purple-spotted bigeye exhibits promising attributes, aligning with commercial standards and highlights the potential of fishery by-products as a sustainable and halal source of gelatine.

**Highlights**

- The pre-treatment involving hydrochloric acid (HCl) immersion with a concentration of 0.25 M demonstrated the highest weight.
- The hydroxyproline analysis showed an insignificant increase (0.088 mg/mL−0.103 mg/mL) from the pre-treatment stage to the final gelatine product.
- The physicochemical properties of the liquid gelatine, including yield (6.5 ± 0.39%), pH (6.55 ± 0.11) and gel bloom strength (174 ± 8.54 blooms) conformed to Gelatin Manufacturers Institute of America (GMIA). Functional groups confirmed the presence of gelatine-specific, such as amides A, B, I, II and III. The molecular profile is comparable to commercial gelatine, with α1 chains at 130 kDa, α2 chains at 115 kDa and β chains at 235 kDa.

## INTRODUCTION

Protein is an important biopolymer that can be obtained from animals, serving as an abundant source of nutrients necessary for body growth and development (Rehman *et al*. 2019). Fish stands out as one of the most abundant and diverse sources of animal protein. This abundance supports the development of the fish processing industry. Around 70% of the industry is processed, with the main focus on fish meat, such as the fish fillet, ground fish meat and surimi industries (Ghaly *et al*. 2013). The processing industry produces by-products in the form of skin, scales, bones and fins, which have the potential to be processed into value-added products. Numerous studies have reported that these fish processing by-products can be utilised to produce derivative products with biofunctional substances (Ortizo *et al*. 2023; Baco *et al*. 2022), serve as sources of fat (Kandyliari *et al*. 2020), minerals (Flammini *et al*. 2016), and protein (Osiriphun *et al*. 2022; Rachman *et al*. 2023). Such utilisation enhances the added value of these by-products.

Fish skin contains type I collagen in the form of fibrillar fibres which provide strength and elasticity to the skin (Coppola *et al*. 2020). Fish scales are also a unique biomaterial consisting of type I collagen and hydroxyapatite (Qin *et al*. 2022). These materials have the potential to be used as raw materials for collagen products. Collagen molecules contain high amounts of three amino acids: Glycine (Gly), proline (Pro) and hydroxyproline (Hyp). The hydroxyproline content is very important for assessing collagen quality because it plays an important role in structural stability and gel strength. Collagen, through the hydrolysis process, will produce gelatine products. However, the presence of minerals in fish skin and scales can inhibit the hydrolysis process of collagen into gelatine. Therefore, it is essential to implement a pre-treatment process to dissolve the minerals. Pre-treatment with hydrochloric acid (HCl) is a widely utilised method, as it effectively dissolves minerals without leaving harmful residues. However, it is crucial to carefully control the concentration and duration of the pre-treatment process to prevent damage to hydroxyproline and to preserve the quality of the gelatine (Lueyot *et al*. 2021). In addition, the gelatine extraction process must be carried out under optimal conditions, such as maintaining a temperature between 50°C to 100°C, to prevent protein degradation and maintain gel strength and gelatine viscosity (Nurilmala *et al*. 2022).

The unique physicochemical properties of gelatine have led to its wide application in various fields, serving as a stabiliser, thickener and emulsifier in food, pharmaceuticals and cosmetics (Azilawati *et al*. 2015). Gelatine products currently in circulation generally use raw materials sourced from pigs, cowhide, beef bones and fish, contributing 41%, 28.5%, 29.5% and 1% of the total world production, respectively (Ali *et al*. 2016; Milovanovic & Hayes 2018). The high production of collagen and gelatine from pigs and cows raises concerns among adherents of certain religions. It is forbidden for Muslims to consume pork and some animals with slaughter processes that do not adhere to Sharia guidelines. Similarly, it is prohibited for Jews and Hindus to consume cows (Rakhmanova *et al*. 2018). Additionally, raw materials from bovine animals are associated with bovine spongiform encephalopathy (BSE), commonly known as mad cow disease disease (Forooghi *et al*. 2023).

Fish gelatine, as a raw material, still shows minimal production while being classified as the safest raw material. It does not pose problems related to halal guidelines or specific diseases, and the availability of abundant and varied raw materials continues to be explored. Recent studies, such as those by (Widiyanto *et al*. 2022) have highlighted the high hydroxyproline content found in the scaly skin of demersal fish, particularly the purple-spotted bigeye fish (*Priacanthus tayenus*). Research on this topic has predominantly focused on using fish skin for collagen extraction (Oslan *et al*. 2022) and gelatine characterisation (Sukkwai *et al*. 2011). However, studies used explicitly of purple-spotted bigeye fish scaly skin (PSSS) is still limited. Hence, this research is essential to evaluate the optimal conditions for mineral removal during pre-treatment, analyse hydroxyproline content at various stages of processing and characterise the gelatine product.

## MATERIALS AND METHODS

### Purple-Spotted Scaly Skin Preparation

PSSS, obtained from the fish fillet industry at Tegalsari Beach Fishing Harbour, Tegal City, Indonesia, was meticulously cleaned and washed to remove residual meat, fat and bones. The fish used in this process had 20 cm–23 cm length, with an average weight of 181.8 g. The skin and scales comprised ±8.49% of the total fish weight. The cleaned PSSS was then cut into small pieces (±1 cm^2^) and placed in polyethylene plastic bags. The prepared samples were subsequently stored at −20°C until they were required for further processing.

#### Pre-treatment

The first step of pre-treatment was carried out following the method of Kittiphattanabawon *et al*. (2010) with a modification. The sample was immersed in 0.1 M NaOH with a ratio of 1:10 (w/v) for 16 h at room temperature (25°C–28°C). Subsequently, the samples were washed with running water until the skin reached a neutral pH.

The next step involved mineral removal using HCl solutions at concentrations of 0.25 M, 0.5 M, 0.75 M and 1 M, at a ratio of 1:10 (w/v) for 1 h with continuous stirring at 25°C–28°C. After treatment, the samples were rinsed with running water until a neutral pH was reached. Samples treated with 0.25 M HCl were selected for subsequent processes due to their optimal results in mineral removal.

#### Swelling

The selected samples treated with 0.25 M HCl were then subjected to the swelling process based on a modified method from (Nurilmala *et al*. 2021). These samples were immersed in a 0.2% citric acid solution for 12 h, with a sample-to-solution ratio of 1:4. The purpose of the citric acid immersion was to facilitate the development of collagen material in the sample matrix. After immersion, the samples were thoroughly rinsed until a neutral pH was achieved. The swelling degree (%) was calculated using the following formula:


$$\text{Swelling degree }(\%)=\frac{\text{Weight of swollen polymer}-\text{Weight of polymer}}{\text{Weight of polymer}}\times 100\%$$

### Extraction of Gelatine

Samples that had undergone the swelling stage were extracted using a water bath shaker at 65°C for 7 h, with a PSSS and distilled water ratio of 1:1 (w/v). The mixture was then filtered using calico cloth, and the liquid gelatine obtained was dried using an oven at 50°C for 24 h. The resulting dried gelatine was used for yield calculation. The calculation of the gelatine yield referred to AOAC (1995), which is the ratio of the dry weight of gelatine to the specific gravity of fresh skin (a type of skin that has been cleaned of meat, fat and other impurities). The yield value can be calculated using the following formula:


$$\text{Gelatine yield }(\%)=\frac{\text{Gelatine weight}}{\text{Fresh skin weight}}\times 100\;\%$$

### Analysis of Hydroxyproline Content

The measurement of hydroxyproline content followed the procedure outlined by Sigma-Aldrich (2021). The samples analysed for hydroxyproline included the fish skin that had undergone pre-treatment, swelling and the liquid gelatine. The analytical procedure began with weighing a 10 mg sample into a heat-resistant container (sample tube), followed by homogenisation using a tissue grinder pestle. The sample was then dissolved in 100 μL of dH_2_O and 100 μL of concentrated HCl (~12N) was added. The sample tube was tightly closed and covered with aluminum foil. Subsequently, it was hydrolysed at 120°C for 3 h, centrifuged using a vortex at 10,000 × *g* for 3 min and 10 μL of supernatant was transferred to a well plate. In determining the hydroxyproline content, a standard series was first prepared. Ten microliters (10 μL) of standard hydroxyproline solution were dissolved with 90 μL of dH_2_O to make a 0.1 mg/mL standard solution. This solution was then moved in varying amounts (0 (blanco), 2 μL, 4 μL, 6 μL, 8 μL and 10 μL) into the well plate, producing Hydroxyproline Standard (Sigma-Aldrich, St. Louis, MO, USA) at concentrations of 0, 0.2, 0.4, 0.6, 0.8 and 1 μg/well standards.

The assay involved a mixture of chloramine-T oxidation buffer and 4-dimethylamino benzaldehyde (DMAB) reagent. A 100 μL mixture of chloramine-T oxidation buffer assay was prepared by combining 6 μL of chloramine-T and 94 μL of oxidation buffer into each well containing the sample and standard hydroxyproline. The mixture was incubated at room temperature for 5 min. Subsequently, a 100 μL DMAB reagent assay was prepared by mixing 50 μL of DMAB concentrate and 50 μL of perchloric acid/isopropanol solution into each well containing the chloramine-T oxidation buffer assay, sample and standard hydroxyproline. The mixture was then incubated using a dryer, baked for 90 min at 60°C, and the absorbance at 560 nm was read on a microplate reader or ELISA reader (Thermo Fisher Scientific, MS, USA). Measurements were repeated twice (Duplo). The formula for calculating the concentration of hydroxyproline is as follows:


$$\text{Concentration of hydroxyproline}=\frac{\text{Total hydroxyproline from standard curve }(\mu \text{g})}{\text{Sample volume added to reaction well }(\mu \text{L})}$$

### Viscosity Analysis

The viscosity of the gelatine solution was measured according to Mohtar *et al*. (2010), with a slight modification. A total of 6.67 g of gelatine powder was weighed and dissolved in 100 mL of distilled water in an Erlenmeyer flask, resulting in a solution of 6.67% w/v, at room temperature for 30 min. The solution was then incubated in a water bath at 60°C until completely dissolved. The viscosity of the dissolved gelatine was measured at 60°C in a beaker using a viscometer (RDT-CPS, AMETEK Brookfield, Berwyn, PA, USA) at 50 rpm (Nurilmala *et al*. 2020).

### Gel Strength Analysis

Gelatine powder, weighing 7.5 g, was dissolved in 150 mL of distilled water to form a gelatine solution (6.67%, w/v). This solution was then placed in a bloom bottle, covered with aluminum foil and incubated in a water bath at 50°C for 4 h or until homogeneous. Subsequently, the gelatine solution was left at room temperature until its temperature approached room temperature, followed by cooling at 10°C for 3 h until the gelatine solution solidified. Bloom or gel strength was measured using a GCA geometer (Precision Scientific Group, Chicago, IL, USA). The unit indicating the strength of the gel resulting from a certain concentration is called the degree of bloom.

### pH Measurement of Gelatine

The pH measurement followed the method outlined by Schrieber and Gareis (2007) with modifications by Nurilmala *et al*. (2017). In this process, 0.75 g of gelatine powder was dissolved in 50 mL of distilled water in a glass beaker, heated in a water bath at 50°C until homogeneous. The gelatine solution was then cooled to room temperature and measured using a pH meter (Metrohm, Switzerland) that had been calibrated with pH 4 and pH 7 buffers at 35°C. The electrode was immersed in the gelatine solution for a few moments until a stable reading was recorded on the pH meter monitor.

### Functional Group Analysis by Fourier Transform Infrared Spectrometry

Analysis was conducted to determine the functional groups in the resulting gelatine, following the method outlined by (Muyonga *et al*. 2004). A 2 mg gelatine sample was ground with KBr (Sigma-Aldrich, St. Louis, MO, USA) in a mortar until homogeneous. The resulting mixture was placed into a pellet mold, compacted and vacuumed using a pellet molding machine. The pellet was then inserted into the cell and positioned in the cell placement chamber. Subsequently, it was irradiated with IR light from the IR-408 infrared spectrophotometer, which had been turned on under stable conditions.

### Molecular Weight Analysis

Analysis of the protein profile of the scaly skin gelatine of the purple-spotted fish was carried out using Sodium Dodecyl Sulphate Polyacrylamide Gel Electrophoresis (SDS-PAGE), following the method by Laemmli (1970) modified by Nurilmala *et al*. (2017). The analytical procedure began with sample preparation by dissolving it in SDS, an ammonia detergent, with a ratio of 1:1 (v/v). The sample was homogenised using a mortar and stamper, then heated for 1 h using a dry block thermostat at 85°C.

The gel used consisted of a 12.5% separating gel and a 3% stacking gel. The mixture of the separating gel and stacking gel is referred to as the polyacrylamide gel. The analysis procedure initiated with the insertion of 5 μL of the prepared sample and the protein marker into the polyacrylamide gel. Electrophoresis was conducted constantly at 10 mA current and 125 V electric voltage for 3 h. SDS-PAGE detection was performed by removing the electrophoretic gel from the mold, followed by staining the gel in a staining solution with a composition of 10% glacial acetic acid, 50% methanol, 0.055% CBB and 40% distilled water. Immersion in the staining solution for 1 h was carried out by stirring using a shaker.

The next stage involved destaining to wash or remove colour from the gel. The destaining solution was prepared with a composition of 10% glacial acetic acid, 50% methanol and 40% distilled water. The destaining process continued until the protein bands were clearly visible and the molecular weight was analysed using Photocapt software (Vilber, USA).

### Statistical Analysis

The research design for the pre-treatment step mainly to mineral removal stage and the stage of determining the effect of hydroxyproline content on the gelatine extraction process used a Completely Randomised Design (CRD) and was analysed by the analysis of variance (ANOVA) method, utilising SPSS 22 software (International Business Machines, USA).

## RESULTS

### Skin Weight After Pre-treatment

**Figure 1:**
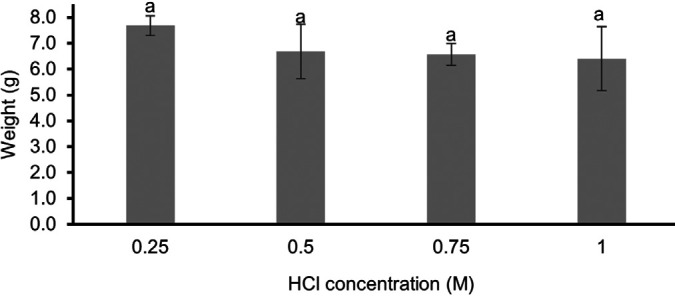
Skin weight after pre-treatment using HCl immersion with different concentrations. Different superscripts indicate significant differences (*p* < 0.05).

The pre-treatment process involved soaking PSSS in NaOH to remove minerals and soften the tissue by immersing it in HCl at various concentrations. This step was intended to eliminate calcium and other inorganic substances, resulting in a softer ossein structure to facilitate extraction. Fig. 1 presented the ANOVA results, showing that varying HCl concentrations did not significantly impact the weight of the PSSS (*p* > 0.05). Therefore, the sample treated with 0.25 M HCl, identified as the most cost-effective concentration for demineralisation, was selected for further analysis to ensure the process’s effectiveness without compromising the quality.

### Swelling Degree

The use of citric acid in hydrolysis was intended to modify the collagen’s fibrous structure, causing it to swell, thus facilitating the extraction process. The swelling degree of the scaly skin in the citric acid solution was measured at 156 ± 4%, indicating a significant increase in volume due to the hydrolysis process.

### Hydroxyproline Content

**Figure 2:**
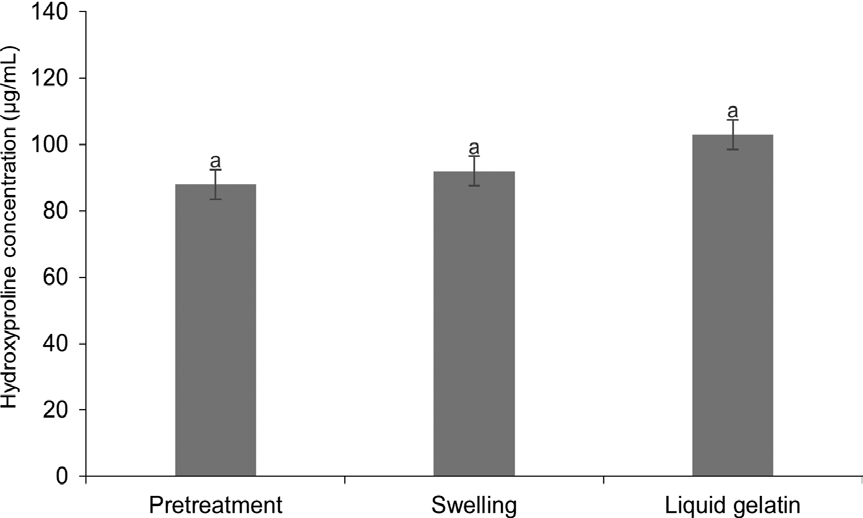
Hydroxyproline content in the stages of production the gelatine PSSS. Different superscripts indicate significant differences (*p* < 0.05).

The hydroxyproline concentration at various stages of gelatine production, including pre-treatment, swelling and the final liquid gelatine, is presented in Fig. 2. The results show concentrations of pre-treatment (88.0 μg/mL), swelling (92.0 μg/mL) and liquid gelatine (103.0 μg/mL). Although there are slight variations, the ANOVA results indicate no significant differences (*p* > 0.05). This suggests that the hydroxyproline content remains relatively stable throughout the processing stages, maintaining consistent gelatine quality.

### Function Group of Gelatine

**Figure 3:**
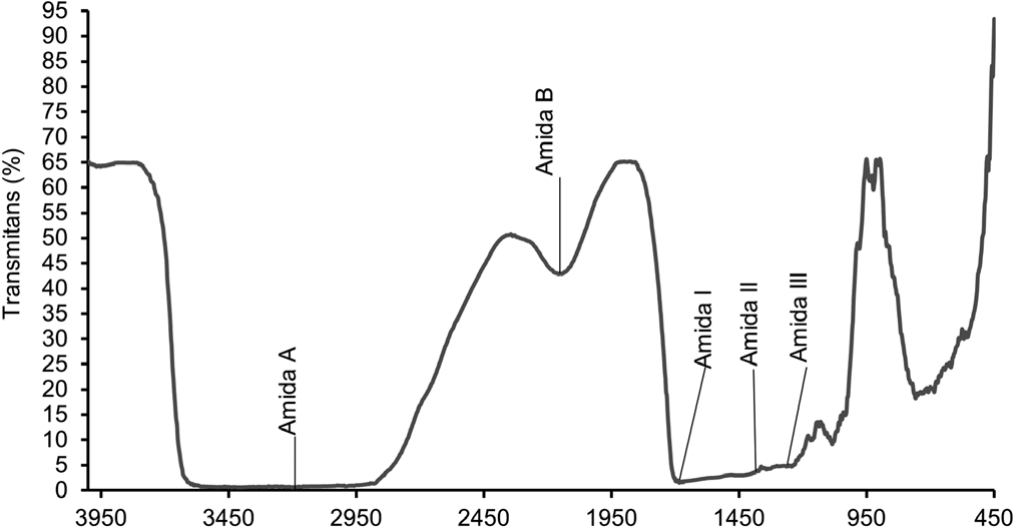
Spectrum FTIR of gelatine PSSS.

The Fourier Transform Infrared (FTIR) spectrum of PSSS gelatine shown in Fig. 3 displays characteristic peaks associated with various functional groups present in gelatine, such as Amide A, B, I, II and III, which are related to the triple-helix structure of collagen or gelatine. This spectrum confirms that the produced gelatine contains the main structures and components consistent with the typical characteristics of gelatine.

### Properties of Gelatine

The physicochemical properties of the gelatine, including yield, pH, viscosity and gel strength, were measured and compared to the international standards set by the GMIA. These parameters are crucial for determining the quality and suitability of gelatine for various applications (Table 1).

### Gelatine Molecular Weight

**Figure 4:**
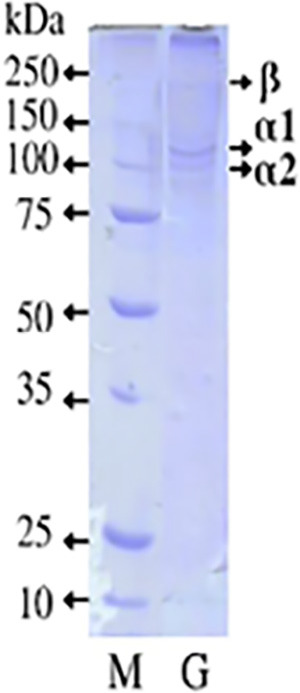
MW of Marker (M) and gelatine of PSSS (G).

Fig. 4 presents the molecular weight (MW) distribution analysis of gelatine extracted from PSSS. The visible bands in the PSSS gelatine indicate the presence of α1 chains at 130 kDa, α2 chains at 115 kDa, and β chains at 235 kDa. These bands are characteristic of gelatine, reflecting the partial hydrolysis of collagen into individual polypeptide chains. This distribution is crucial for understanding the functional properties of the resulting gelatine.

## DISCUSSION

The HCl solution can remove organic matter on fish scaly skin by dissolving calcium phosphate into phosphoric acid. The acid concentration can vary depending on the nature of the material, temperature and time of demineralisation, including the ratio of material to solution (Feng *et al*. 2015). Chuaychan *et al*. (2016) stated that fish scales contain hydroxyapatite and calcium carbonate (CaCO_3_), the surface layer of fish scales is a layer of bone consisting of collagen fibrils in mineral form.

The optimal treatment for mineral removal in PSSS scaly skin was identified with a HCl concentration of 0.25 M. Following the demineralisation stage, a significant portion of calcium (Ca) and phosphorus (P) was effectively eliminated, leading to an increase in the organic matter content, including carbon (C), nitrogen (N) and oxygen (O) in the sample. This specific treatment not only ensured the efficient removal of mineral components but also optimally prepared the material for subsequent processing. The chosen concentration was deemed ideal for facilitating the following steps in gelatine extraction, ensuring the scales were sufficiently demineralised and ready for further conversion into gelatine (Chuaychan *et al*. 2016).

According to Rýglová *et al*. (2021) the acid or alkaline agents destroy the hydrogen bond and cleave a number of covalent bonds which then destabilise the triple helix, thus resulting in undesirable conversion and an increase in the proportion of gelatine. Furthermore, during the immersion process in citric acid, collagen fibrils undergo a swelling process resulting in a decrease in the internal cohesion of the collagen fibres. During swelling, the triple-helix structure of the collagen molecule becomes exposed. Protons that enter the skin structure either lose minerals, or there is a space in tropocollagen, which serves as the entry point for H^+^ ions from acids. H^+^ ions interact with carboxyl groups to change the inter- and intramolecular tropocollagen bonds (Karlina & Atmaja 2010). Citric acid acts as a catalyst between bonds, allowing water to enter the collagen fibril spaces, leading to the swelling of the PSSS that has undergone immersion. This interaction is indicated by an increase in the weight of the PSSS after immersion for a certain period.

The hydroxyproline content of the raw material for PSSS was 145 ± 0.00 μg/mL Widiyanto *et al*. (2022), and after pre-treatment, it was 88.0 μg/mL. After immersion in citric acid, the content was 92.0 ± 0.02 μg/mL, and in the liquid gelatine, it was 103.0 ± 0.02 μg/mL. The results of this study were higher than the concentration of hydroxyproline from tilapia skin collagen (*Oreochromis niloticus*), extracted using acetic acid, which was 23 μg/mL (Reátegui-Pinedo *et al*. 2022). The analysis results of the hydroxyproline content showed that there was no significant increase in the content due to the acid pre-treatment, swelling and gelatine extraction. Swelling treatment on collagen can strengthen the hydrogen bonds that stabilise the triple helix structure of the collagen molecule, thereby increasing the hydroxyproline content in gelatine. This swelling process helps to open the helical structure, which allows the release of more hydroxyproline, which is important for the quality and stability of gelatine (Rýglová *et al*. 2021). Research by (Samatra *et al*. 2024) reported that during the swelling process of buffalo bone gelatine using 0.05 M citric acid, a decrease in hydroxyproline concentration was observed, attributed to collagen degradation. Therefore, careful control of pre-treatment conditions is essential to produce high-quality gelatine with optimal hydroxyproline concentration. In addition, the gelatine extraction process has a significant impact on hydroxyproline retention. According to Atma *et al*. (2018), gelatine extraction requires optimal temperature conditions (60°C–65°C) to maintain hydroxyproline. This temperature range is sufficient to break down collagen bonds without reducing the hydroxyproline content.

The gelatine yield varies for each type of fish skin and scales because they contain different amounts of collagen. The higher the collagen content in the raw material, the more gelatine can be extracted. The yield of PSSS gelatine is 6.5 ± 0.39%. This result is higher than the yield of gelatine from milkfish scales, which was 3.0% (Samosir *et al*. 2018), while the yield of gelatine from cartilaginous fish (*elasmobranch*), a ray-like fish living in demersal waters, was 8.16 ± 0.75% from the skin (Agustini *et al*. 2020). These results align with those reported by Widiyanto *et al*. (2022), where the chemical composition of the skin and scaly skin has a higher protein content than scales, mainly due to a higher ash content that affects the resulting gelatine yield. Another factor influencing gelatine yield is the concentration of the HCl solution used in the mineral removal stage. The higher the concentration, the more acidic the solution, leading to increased hydrolysis of collagen from the triple helix chain into a single chain by many H+ ions. A higher solution concentration is believed to decrease the yield value of the resulting gelatine. The elevated HCl concentration also affects the ossein in the scaly skin of the purple-spotted fish produced. The greater the mass of ossein obtained, the higher the percentage of gelatine yield, as more mass of ossein leads to more collagen breaking down into gelatine (Fernianti *et al*. 2020).

The pH value of gelatine depends on the process used. Acidic processes tend to produce low pH values and alkaline processes tend to yield high pH values. The acid-immersion process causes the collagen fibrils in the skin to swell, reducing the internal cohesion properties of the skin fibres. Swelling results in the amino acid bond structure opening in the collagen molecule, trapping acid in the collagen fibril network. This trapped acid does not dissolve during the neutralisation process and is carried away during the extraction process, impacting the acidity level of the gelatine. Research by Zhou and Regenstein (2005) suggests that a combination of alkaline pre-treatment followed by acid pre-treatment in gelatine manufacturing produces a pH close to neutral for extraction. Gelatine with a neutral pH is generally preferred, making the neutralisation process crucial in neutralising the remaining acid after immersion (Oktaviani *et al*. 2022). Based on the research results, the pH of PSSS gelatine obtained was 6.55 ± 0.11, a value in accordance with the standard of the Gelatin Manufacturers Institute of America (GMIA 2019).

Determination of the quality of gelatine is based on the analysis of its viscosity value, in addition to the gel strength analysis. Viscosity measurements are conducted to assess the level of gelatine viscosity in solution form at a specific concentration, temperature and rotation speed, then converted to millipoise (GMIA 2019). From the analysis results, the viscosity value of the resulting PSSS gelatine is 15.57 ± 1.35 cP. This value is higher than the GMIA standard (2019) of 1.5 cP–7.5 cP. It is believed that the immersion time in the mineral removal process, which was not too long (1 h of HCl solution immersion), contributed to this high viscosity value. A longer immersion time tends to result in a lower viscosity value due to the breakdown of amino acid chains in the gelatine, causing the chains to become shorter (Febriana *et al*. 2021).

The hydroxyproline content in gelatine is critically important for determining its gel strength, a key physicochemical parameter that defines the functionality of gelatine, particularly as an emulsifier and gelling agent (Lueyot *et al*. 2021). Gel strength measures gelatine’s ability to transition between gel and sol phases, which is advantageous due to its reversibility, allowing for versatile applications in food and non-food products. Recent analysis has shown that the gel strength of gelatine derived from PSSS is ± 8.54 blooms, closely aligning with the GMIA standard for food-grade gelatine, which requires a minimum of 175 blooms (GMIA 2019). Several factors influence gel strength, including raw materials, pre-treatment and extraction conditions, amino acid chain length, pH, molecular weight and temperature during gelation (Febriana *et al*. 2021; Zhou & Regenstein 2021). Notably, longer amino acid chains contribute to greater gel strength by allowing more extensive cross-linking within the gelatine matrix, resulting in a stronger gel (Badii & Howell 2006). Optimising these factors, particularly hydroxyproline content, could further enhance gelatine’s gel strength, making it more suitable for a broader range of applications (Gómez-Guillén *et al*. 2002).

FTIR analysis was carried out to ensure that the resulting compound was gelatine by comparing the results of the sample spectra (Maryam *et al*. 2019). According to Rohman *et al*. (2011), FTIR analysis only requires easy preparation with the use of solvents and chemical reagents that can produce fast and low-impact analysis. It is a quick analysis technique that does not cause damage or failure. The results of the FTIR spectrum show four peaks in the wave number region, namely wave numbers 3,595 cm^−1^−2,879 cm^−1^; 2,156 cm^−1^; 1,696 cm^−1^−1,668 cm^−1^; 1,373 cm^−1^−1,343 cm^−1^; 1,297 cm^−1^−1,244 cm^−1^, representing the amide A, amide B, amide I, amide II and amide III regions, respectively, as stated by Shahvalizadeh *et al*. (2021). The analysis of functional groups in gelatine from PSSS revealed the absorption peak of amide A identified in a broad wave between 3,595 cm^−1^−2,879 cm^−1^, indicating the presence of the OH group of hydroxyproline. The broad peak shape is evidence of the OH group of hydroxyproline. The N-H group of a peptide involved in hydrogen bonding might overlap with the O-H group in that area, causing an absorption with a broad peak.

The amide B portion, with absorption at 2,156 cm^−1^, suggests that the N-H group in the amide tends to bond with the CH2 strain when the carboxylic group is in a stable state. This indicates that the samples analysed by FTIR have OH groups, NH strains and CH_2_ strains. The amide I region, with absorption at 1,696 cm^−1^−1,668 cm^−1^, indicates the presence of the C=O strain and the OH group paired with the carboxyl group. The PSSS gelatine shows an absorption region at 1,696 cm^−1^−1,668 cm^−1^, indicating a random coil structure, which is a typical gelatine group. Amide II, with absorption at 1,373 cm^−1^−1,343 cm^−1^, is caused by the deformation of NH bonds in proteins and C=N stretching vibrations. It is associated with the deformation of tropocollagen into α-helix chains, resulting from the denaturation process using heat. The amide III region, with low intensity and almost invisible due to the loss of the collagen triple helix structure during denaturation into gelatine, was detected in the absorption region at 1,297 cm^−1^−1,244 cm^−1^. This indicates the transformation from the triple helix structure to the random coil structure during the extraction process, a result of collagen denaturation into gelatine.

Gelatine generally consists of α chains and β chains as the main proteins. The MW profile of the gelatine protein from PSSS revealed bands of α1 chains at 130 kDa, α2 at 115 kDa and β chains at 235 kDa. Gelatine typically has a high molecular weight, ranging between 80 kDa and 250 kDa. It generally comprises a single α chain, two covalently cross-linked α chains referred to as β chains, and three covalently connected α chains known as γ chains (Haug *et al*. 2004). The α chain’s reported MW falls between 80 kDa–125 kDa, the β chain between 160 kDa–250 kDa, and the γ chain between 240 kDa–375 kDa (Gudipati 2013). In the SDS-PAGE profile of PSSS gelatine, a band above 250 kDa is suspected to be a γ chain, suggesting covalent connections of three α chains and the visibility of the β chain. This indicates the presence of cross-links in the molecule, formed by covalently linked α chain dimers. Some factors contributing to differences in MW of gelatine include species variations; for instance, warm-water fish exhibit higher MW components than cold-water fish (Gómez-Guillén *et al*. 2002). The extended storage of raw materials in a frozen state can also impact the MW of gelatine due to protein denaturation, as observed by Silva *et al*. (2014), with the MW profile of cobia (*R. canadum*) and croaker (*M. furnieri*) gelatine α1 chain at 166 kDa. Factors such as acid pre-treatment and extraction temperature influence the protein structure of gelatine (Zuraida & Pamungkas 2020), potentially causing more severe damage to the collagen structure. It is suspected that citric acid breaks peptide bonds, resulting in reduced MW (Niu *et al*. 2013), although citric acid is effective in removing phospholipids and plays a crucial role in binding amino acid residues to collagen (Benjakul *et al*. 2009). The MW distribution component of seawater fish is higher than that of freshwater fish, possibly because seawater fish have more collagen components than freshwater fish.

## CONCLUSIONS

The pre-treatment involving HCl immersion with a concentration of 0.25 M demonstrated the highest weight. PSSS, a by-product of the fisheries industry, can be utilised as an ingredient in gelatine production due to its hydroxyproline content, which serves as an indicator of collagen in the gelatine-making material. The hydroxyproline analysis showed an insignificant increase (0.088 mg/mL−0.103 mg/mL) from the pre-treatment stage to the final gelatine product. The obtained liquid gelatine product exhibited yield, pH and gel strength in accordance with GMIA standards. It displayed a molecular weight band with an α1 chain at 130 kDa, α2 at 115 kDa and β chain at 235 kDa. The functional groups identified included amides A, B, I, II and III, indicating the presence of gelatine products and their derivatives.

## Figures and Tables

**Table 1:** Physicochemical parameters of PSSS gelatine.

Parameters	PSSS gelatine	GMIA standards (2019)
Yield	6.5 ± 0.39%	–
pH	6.55 ± 0.11*	4.5–6.5
Viscosity	15.57 ± 1.35 cP	1.5–7.5 cP
Gel strength	174 ± 8.54 bloom*	150–300 bloom

*Note*: Data are presented as means ± SD (*n* = 3. The marker (*) indicates value according to GMIA standard (2019)
